# Selection on the Major Color Gene Melanocortin-1-Receptor Shaped the Evolution of the Melanocortin System Genes

**DOI:** 10.3390/ijms18122618

**Published:** 2017-12-05

**Authors:** Linda Dib, Luis M. San-Jose, Anne-Lyse Ducrest, Nicolas Salamin, Alexandre Roulin

**Affiliations:** 1 Department of Ecology and Evolution, Biophore, University of Lausanne, 1015 Lausanne, Switzerland; linda.dib@unil.ch (L.D.); luis.sanjosegarcia@unil.ch (L.M.S.-J.); anne-lyse.ducrest@unil.ch (A.-L.D.); 2 Laboratoire de Recherche en Neuroimagerie, Centre Hospitalier Universitaire Vaudois, 1015 Lausanne, Switzerland; 3 Swiss Institute of Bioinformatics, Quartier Sorge, 1015 Lausanne, Switzerland; 4 Department of Computational Biology, University of Lausanne, Rue du Bugnon 27, 1011 Lausanne, Switzerland

**Keywords:** melanocortin system, pleiotropy, gene evolutionary influence, coevolution, selection

## Abstract

Modular genetic systems and networks have complex evolutionary histories shaped by selection acting on single genes as well as on their integrated function within the network. However, uncovering molecular coevolution requires the detection of coevolving sites in sequences. Detailed knowledge of the functions of each gene in the system is also necessary to identify the selective agents driving coevolution. Using recently developed computational tools, we investigated the effect of positive selection on the coevolution of ten major genes in the melanocortin system, responsible for multiple physiological functions and human diseases. Substitutions driven by positive selection at the melanocortin-1-receptor (*MC1R*) induced more coevolutionary changes on the system than positive selection on other genes in the system. Contrarily, selection on the highly pleiotropic POMC gene, which orchestrates the activation of the different melanocortin receptors, had the lowest coevolutionary influence. *MC1R* and possibly its main function, melanin pigmentation, seems to have influenced the evolution of the melanocortin system more than functions regulated by *MC2-5R*s such as energy homeostasis, glucocorticoid-dependent stress and anti-inflammatory responses. Although replication in other regulatory systems is needed, this suggests that single functional aspects of a genetic network or system can be of higher importance than others in shaping coevolution among the genes that integrate it.

## 1. Background

Characterizing the organization of an organism into autonomous genetic and phenotypic modules has gained strong theoretical and empirical interest [1,2,3,4]. Such modular organization helps to understand the evolutionary dynamics of complex organisms given that the compartmentalization of a function (or of a set of functions) will permit this functional aspect to evolve independently of how selection acts on other functional aspects of the organisms (i.e., it alleviates the so-called “cost of complexity” or “cost of pleiotropy” [5,6,7]). However, understanding the evolution of such functional modules requires a characterization of the interactions between the different elements within a module and how these interactions determine their joint evolution [1,2]. Recently-developed computational tools can complement our understanding of molecular evolution within a genetic system or network by estimating the probability that a nucleotide site coevolved with a site in another gene across a phylogenetic tree [8,9]. The use of such tools is a major difference in relation to previous studies, where only the coevolution within a protein or gene sequence (e.g., due to structural constraints) could be considered [10].

**Figure 1:**
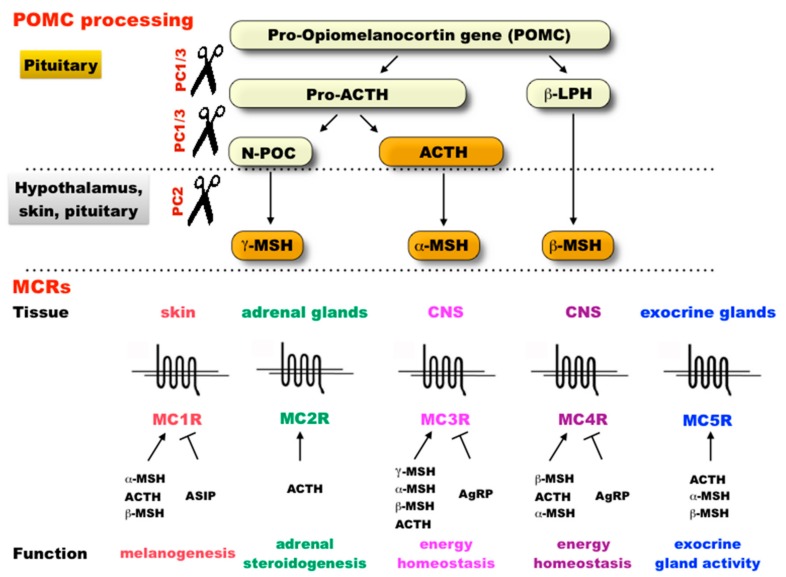
Schematic presentation of the melanocortin system in human. The melanocortins (ACTH, α-, β- and γ-MSHs) are peptide hormones derived from the proopiomelanocortin (POMC) prohormone through tissue-dependent post-translational modification by two convertases: PC1/3 (encoded by *PCSK1*) cleaves the prohormone to obtain ACTH and β-LPH, and cleavage of ACTH by PC2 (encoded by *PCSK2*) gives α-MSH. β- and γ-MSH are also obtained by PC2 cleavage of POMC. Together with the antagonists, agouti-signalling protein (ASIP) and agouti-related protein (AgRP), the melanocortins bind to five melanocortin receptors MC1-5Rs with various affinities. MC1R mainly regulates melanogenesis, whereas the specific ACTH-receptor MC2R is essential for the regulation of glucocorticoidogenesis. MC3-5Rs are involved in the regulation of energy homeostasis (food intake, energy storage, lipolysis), autoimmune response, anti-inflammatory, cardiovascular and natriuretic processes, sexuality and social behavior. See references in the main text. In the figure, only the main tissues and functions in human were noted for simplification.

The melanocortin system is a key hormonal pathway that exhibits the features of a module. It is composed of a set of G protein-coupled membrane receptors (MC1-5R) responsible for the regulation of very distinct functions in vertebrates: from pigmentation to metabolic homeostasis and sexual behavior (Figure 1) [11,12,13,14,15,16,17]. The activity of the different receptors and their associated functions is under the control of a shared set of agonist and antagonist ligands. The upregulation of the distinct receptors is achieved by binding one of the melanocortin hormones that are derived from the same prohormone: the pro-opiomelanocortin, POMC (Figure 1) [14,15,18]. The proprotein convertase, PC1/3, encoded by the gene *PCSK1*, cleaves the POMC prohormone into the adrenocorticotropic hormone, ACTH, which, in turn, can be cleaved by the convertase PC2 (*PCSK2*) to release the melanocortin α-MSH (Melanocyte Stimulating Hormone [18,19]. β-MSH as well as other melanocortins (γ-, and δ-MSH) are present only in certain vertebrate taxa [11] and are also derived from POMC by the cleaving action of PC1 and PC2 [20]. Activation of a melanocortin receptor has several cascading effects by raising intracellular cAMP levels [21]. In contrast, down-regulation of the MC receptors activity, is triggered by binding the antagonists and inverse agonists: the agouti-signaling protein (ASIP, or ASIP1 the fish orthologue of the mammalian ASIP [17]) and the agouti-related protein (AgRP), in a tissue and melanocortin-receptor dependent manner [22].

The evolution of the different genes in the melanocortin system has been previously studied [11,23,24,25]. However, to what extent the genes in the melanocortin system have influenced each other’s evolution has never been assessed. On the one hand, we can expect genes in the melanocortin system to have coevolved to a certain extent owing to selection for module conformation and/or maintenance [1,7]. In particular, the evolution of the regulatory genes (e.g., *POMC* or *ASIP*) of the melanocortin system may have favored further changes in one or several receptor sequences to induce, for instance, compensatory or co-adaptive effects in relation to the changes in the regulatory gene sequence [26,27]. Changes in a melanocortin receptor may have also favored evolutionary changes in other receptors owing to selection for co-adaptation through the functions controlled by several receptors [26]. On the other hand, other factors, such as pleiotropy, are expected to limit coevolutionary changes [6,28,29]. In this sense, we could expect that the regulatory genes of the melanocortin system, which have more pleiotropic effects by acting on all the different receptors (Figure 1), might have been under higher evolutionary constraints. This might have limited their influence on the evolution of other melanocortin genes and reduce the extent with which coevolution occurred among genes in the system.

Here, we estimated the selective and coevolutionary processes that occurred during the evolution of the melanocortin system. The widespread occurrence and preserved functions of these genes across vertebrates make it a highly suitable system to investigate how and to what extent genes may function within a module coevolve. We focused on the 10 major genes of the melanocortin system (*POMC*, *PCSK1*, *PCSK2*, MC1-5R, *ASIP*, and *AGRP*), whose sequences were obtained for a total of 138 species representing the main vertebrate lineages (16 birds, 2 snakes, 7 lizards, 1 turtle, 1 monotreme, 3 marsupials, 81 placental mammals, 3 amphibians, 1 coelacanth, 20 teleost fish, 2 sharks, and 1 lamprey species: Supplementary Materials Figure S1). We first assessed the evolutionary dynamics of the genes in the melanocortin system by comparing the level of sequence conservation and the proportion of codons evolving neutrally or under positive or purifying selection. This allowed us to understand the genes in the system that are evolutionarily more constrained and to associate this with the coevolutionary dynamics between genes. We then estimated to what extent the genes in the melanocortin system have coevolved by estimating the number of sites that show evidence of correlated evolution [8,9]. We focused on sites within codons evolving under positive selection and inducing coevolutionary changes between genes to better understand the importance that selection had on the distinct genes of the melanocortin system (and possibly on the functional aspects these genes regulate) on determining coevolutionary dynamics within the system.

## 2. Results and Discussion

### 2.1. Molecular Evolution of the Genes in the Melanocortin System

**Figure 2:**
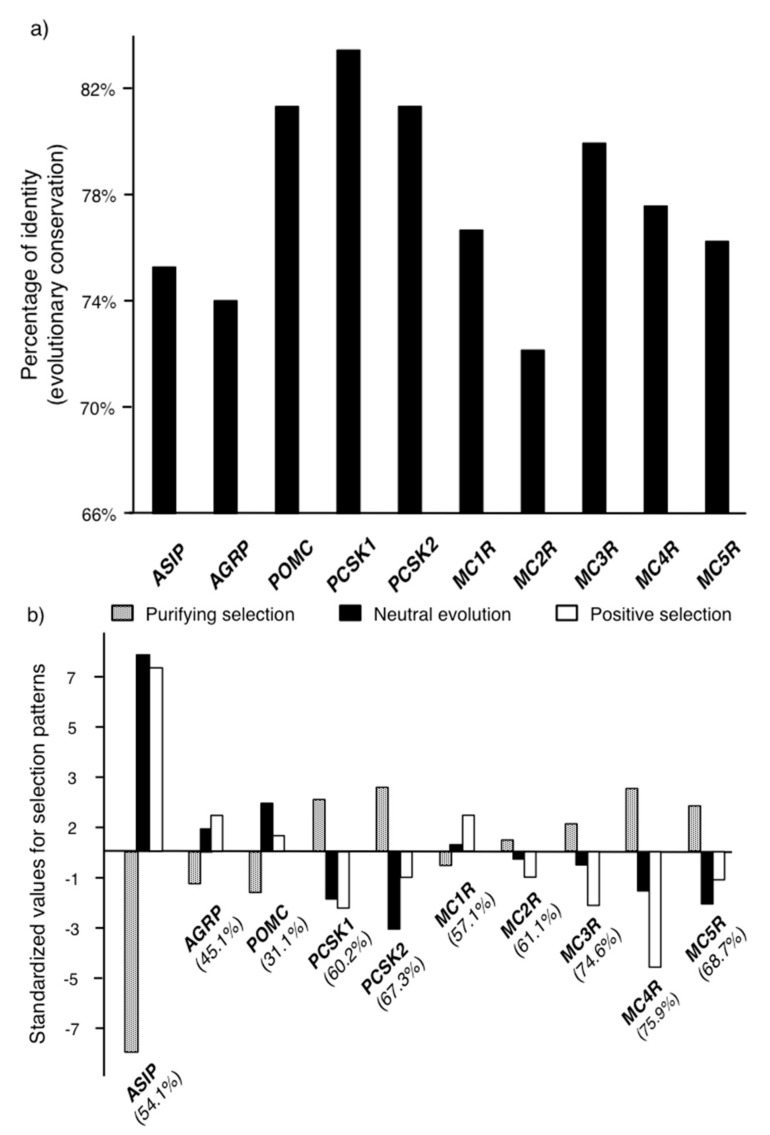
Sequence conservation (**a**) and selection patterns (**b**) among the main genes of the vertebrate melanocortin system. (**a**) Sequence conservation is given by the percentage of identity; (**b**) The percentages of nucleotide sites that experienced positive or purifying selection or that evolved neutrally have been standardized ([value–mean] divided by 1 standard deviation) to facilitate comparisons (see unstandardized data in Supplementary Materials Table S1). The proportion of nucleotide sites that could be unambiguously assigned by MEME to a codon that experienced positive or purifying selection or that evolved neutrally is indicated between parenthesis below the name of each gene.

Genes belonging to the melanocortin system have been traditionally suspected to be highly conserved across vertebrates, although empirical support for this idea has been based on the sequences of a few vertebrate species [11,25]. Using a wider coverage of the vertebrate tree than in previous studies, our results showed that the genes of the melanocortin system have indeed a high level of sequence identity across vertebrates (mean ± SD: 77.8% ± 3.6, Figure 2a). Similar levels of identity have been observed for other genes such as those associated with the regulation of general vertebrate development [30,31] and it is in line with the conserved functions of the melanocortin system across vertebrates [11,13]. Although the differences between genes in sequence identity were not major (maximal difference observed was 12%, Supplementary Materials Table S1), among the most evolutionary conserved genes were the proopiomelanocortin gene (*POMC*) and its two associated convertases, *PCSK1* and *PCSK2* (Figure 2a). This suggests that the genes involved in the production and post-processing of the receptor agonist ligands may be subjected to stronger evolutionary constraints than other genes in the system, which may be the consequence of their higher number of pleiotropic effects [6,13]. However, this contrasts with the fact that the genes encoding the antagonistic ligands (*ASIP* and *AGRP*) were among the least conserved genes, even though they are also expected to mediate some pleiotropic effects by regulating the activity of the different receptors [13,14].

Congruent with the high level of sequence conservation, the MEME analysis indicated that a large proportion of the codons were evolving under purifying selection (mean ± SD 90.7% ± 3.6), whereas a low proportion were under positive selection (2.6% ± 1.6 SD) or neutral evolution (6.7 ± 3.9 Figure 2b, Supplementary Materials Table S1). The proportion of codons under purifying selection across the genes in the melanocortin system was strongly and negatively correlated with the proportion of codons under positive selection (Spearman’s ρ = −0.80, *N* = 10, *P* = 0.005) or evolving neutrally (Spearman’s ρ = −0.95, *N* = 10, *P* < 0.0001). This suggests that the evolution of some genes in the melanocortin system has been less constrained, although all the genes exhibited values within the same order of magnitude and, as suggested by the similar levels of sequence identity, major differences among the genes of the melanocortin system in their evolutionary dynamics do not seem to exist (Supplementary Materials Table S1). The genes exhibiting a higher proportion of codons under positive selection and neutral evolution were those coding for the antagonist receptor ligands: *ASIP*, and *AGRP*, and the *MC1R*. Both, *ASIP* and the *MC1R* are key regulators of melanin synthesis in vertebrates [32,33] and have been observed to mediate adaptive color evolution in several vertebrate species [34,35,36]. Although *ASIP* and, to a minor extent, *MC1R* have functions outside melanogenesis [37,38,39,40], we can hypothesize that vertebrate coloration may be the functional component of the melanocortin system that can be freer to vary and/or have mediated adaptation more frequently than the other functional aspects controlled by the melanocortin system. The *POMC* gene exhibited a relatively high proportion of codons evolving neutrally and the lowest proportion of codons evolving under purifying selection, which contrasts with the relatively higher level of identity of its sequence compared to other genes (Supplementary Materials Table S1). However, it is important to consider that, because of their high degree of conservation among vertebrates, only one third of the *POMC* codons had sufficient statistical signal to be assigned by MEME into one of the three categories of selective pressures (Figure 2b, Supplementary Materials Table S1).

### 2.2. Molecular Coevolution among the Melanocortin System Genes

We estimated coevolution as the joint substitutions between nucleotide sites in two different protein sequences following the simulation procedure described in Dib et al. [9]. Using this method, we estimated the number of significant coevolving nucleotide pairs and, separately, the number of significant coevolving nucleotide pairs that were included in codons under positive selection (Table 1 and Table 2, Supplementary Materials Tables S3 and S4). These sites may be more indicative of adaptive coevolution between genes rather than of constraints due to, for instance, receptor-ligand specificity. Using a ΔAIC threshold representing the 95th percentile (see Methods), the mean (±S.D.) number of coevolving nucleotide pairs was 1071.51 ± 526.7, whereas the mean (±S.D.) number of coevolving pairs with at least one of the nucleotides in the pair being in a codon subjected to positive selection was 181.2 ± 90.23 (Table 1 and Table 2). Using the 90th or the 97.5th percentiles as thresholds resulted in a larger and a smaller number of coevolving sites detected, respectively (Supplementary Materials Tables S3 and S4). Nevertheless, the number of coevolving nucleotide pairs detected for each gene, as well as the number of these coevolving pairs including at least one of the nucleotides within a codon under selection, were highly correlated among the different thresholds used (all Spearman’s ρ > 0.73, all *P* < 0.02).

Consistently across the three thresholds used, the genes that had a significantly higher frequency of coevolving pairs (including nucleotides in codons under selection) compared to all the other genes in the system were the antagonist *ASIP*, *AGRP*, and the receptors *MC1R*, and *MC3R* (Table 1, Supplementary Materials Table S3). The number of coevolving pairs can consider the same sites several times when these have induced evolutionary changes in different sites in another gene (Supplementary Materials Figure S2). When considering only the number of distinct sites in a gene sequence that belongs to a codon under selection and that induced an evolutionary response in other genes, the list of genes highlighted above was narrowed down to *ASIP* and *MC1R* (Table 1, Supplementary Materials Table S3). Those genes with a significantly lower frequency of coevolving pairs (including nucleotides in codons under selection) across all the thresholds were the ligand-related genes *POMC*, *PCSK1*, *PCSK2* and the receptor *MC4R* (Table 1, Supplementary Materials Table S3). When also considering the number of different sites, only *PCSK2* was highlighted using the three different thresholds (Table 1, Supplementary Materials Table S3).

The coevolutionary effects of the genes in the melanocortin system are therefore in line with the differences among these genes at the level of genetic identity and selection. Genes producing agonist ligands (*POMC*, *PCSK1*, *PCSK2*) seem to be more conserved and also to induce less evolutionary changes in other melanocortin genes. Contrarily, genes, such as *ASIP* and *MC1R*, which are less conserved and may have evolved more frequently under selection or by neutral means seem to induce more evolutionary changes in the other melanocortin genes in response to positive selection. This is in part supported by a significant, negative association between sequence identity and the percentage of sites in a gene sequence that induced evolutionary changes in other genes (for all thresholds, Spearman’s ρ > −0.69, all *P* < 0.027). In contrast, the percentage of codons under positive selection, purifying selection, or evolving neutrally was not consistently correlated with any parameters of gene coevolution measured using the different thresholds (the correlation between the percentage of sites in a gene sequence that induced evolutionary changes in another gene and the percentage of sites under positive or purifying selection were significant only when using the less restrictive 90th percentile: Spearman’s ρ = 0.66, *P* = 0.038, ρ = −0.68, *P* = 0.03, respectively). This finding suggests that the selection exerted on individual genes is not associated with the extent to which they influence other genes in the system. However, the relatively low number of genes in the melanocortin system limit the power to assess this confidently using this system. Thus, studies based systems or networks enclosing a larger number of genes may be more suitable in testing to what extent evolutionary patterns of individual genes are linked to their coevolutionary dynamic with other genes.

In addition to measuring the magnitude of coevolutionary effects that a given gene may have towards other genes in the system, it is also relevant to consider the frequency with which such coevolutionary processes have occurred across the vertebrate tree (i.e., the percentage of branches of the vertebrate tree where genes of the melanocortin system coevolved as a result of positive selection in one gene; Supplementary Materials Table S5). We observed that sites under positive selection in the *MC1R* gene have induced coevolutionary changes in other genes more frequently than any of the other genes in the melanocortin system (10.2%, mean across all of the genes of the melanocortin system: 6.3% ± 2.9 S.D., Supplementary Materials Table S5). On the other hand, the lowest frequency (1.5%) was observed for the *POMC* gene. Surprisingly, *PCSK2*, which hosts a low number of coevolving pairs and sites (see paragraph above), has mediated coevolutionary effects with a frequency (9.9%) similar to that of the *MC1R*. This evidences that the coevolutionary action of a gene can be also based on a few sites having influenced the evolution of other genes repeatedly across evolutionary time.

**Figure 3:**
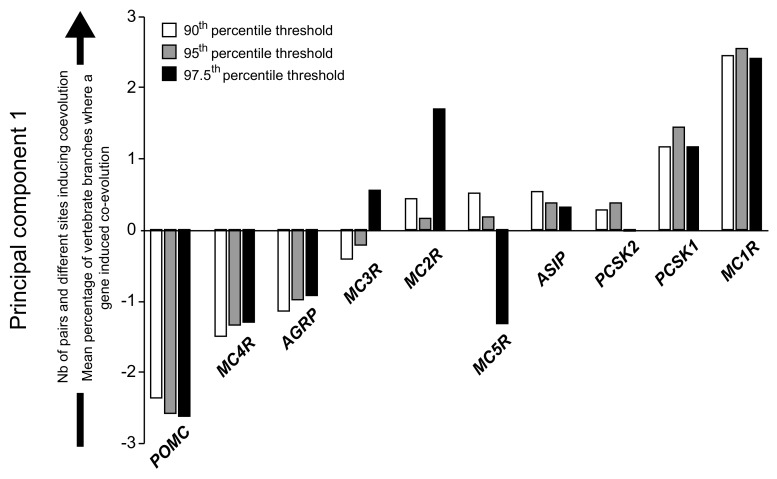
Ordination of genes in the melanocortin system according to their coevolutionary influence onto other genes within the system. Shown are the scores derived from a principal component analysis on the number of pairs containing a nucleotide in a codon under positive selection that induce coevolution in other genes, the number of different sites containing a nucleotide in a codon under positive selection that induce coevolution in other genes, and the percentage of branches in the vertebrate tree where positive selection induced coevolution in other genes. For each gene, we showed the scores using different threshold to detect coevolution between sites.

We applied an ordination method (Principal Component Analysis, PCA) to assess how distant the genes in the melanocortin system are from each other when accounting for the different parameters that assessed their coevolutionary influence onto other genes. We measured this as the number of pairs containing a nucleotide in a codon under positive selection that induce coevolution in other genes, the number of different sites containing a nucleotide in a codon under positive selection that induce coevolution in other genes, and the percentage of branches in the vertebrate tree where positive selection induced coevolution in other genes. For all the thresholds, only the first axis was retained owing to its eigenvalue greater than 1 [41] (explained variance: 58.5, 67.9, 79.6 for the 90th, 95th, and 97.5th percentile thresholds, respectively). For all the thresholds, the variables included in the PCA loaded positively, indicating that positive scores on the PC1 was associated with a higher coevolutionary influence in terms of the number of pairs and different sites inducing coevolution in other genes, and the percentage of branches where a coevolutionary effect of a gene has been observed. All the PC1 derived using the data estimated with the different thresholds ordered *POMC* and *MC1R* as the most negative and positive extremes, respectively (Figure 3). This indicates that, when taking all the parameters into account, *POMC* and *MC1R* can be distinguished as the genes within the melanocortin having less and more general coevolutionary effects, respectively (Figure 3).

### 2.3. Coevolution of the Melanocortin System

Our study provides unprecedented insights into the evolution of a genetic system across vertebrate evolution shedding some light on the evolution of modular organization from a molecular perspective. The analysis of the coevolutionary patterns among the genes of the vertebrate melanocortin system suggests that genes differ to a certain extent on their relative influence towards other genes. Considering the different aspects that determine the coevolutionary influence that sites on a gene subjected to positive selection have on other genes, our study highlights the genes *MC1R* and *POMC* as those having a larger and a smaller coevolutionary influence on other genes of the melanocortin system, respectively. *POMC* is an upstream gene that is key for the regulation of the multiple functions controlled by the melanocortin system [13,25] and it is therefore expected to influence the different elements in the system at several levels. In this sense, the relatively lower coevolutionary influence of the sites at the *POMC* subjected to positive selection towards other genes is somehow surprising. This could be explained by the fact that *POMC* mainly interacts with the other elements of the system through the small and highly conserved melanocortin peptides (Figure 1) [25] and may offer few chances for inducing coevolution at the other genes.

Contrarily to *POMC*, *MC1R* mainly functions in a single context, melanogenesis [42]. Although it is also involved in regulating the inflammatory response [43] and mechanisms of analgesia [39], its main function in regulating the synthesis of melanin pigments allows us to hypothesize that coloration is, among all the functional aspects controlled by the melanocortin system (e.g., anti-inflammatory activity, energy homeostasis, aggressive behaviour, sexual activity, and social behaviour; reviewed in [13]), the one that has been more influential in the coevolution of all the genes in the system. A number of studies have identified different *MC1R* alleles associated with differences in coloration within or between populations of the same species [44], and there is a clear interest in better understanding the evolutionary relevance of the sites harboring these mutations [45,46]. However, it should be noted that our study focused on the long-term evolutionary processes that have led to the divergence of the melanocortin genes across vertebrates and, consequently, it does not account for sequence variation within species. It is therefore unlikely that the same sites would be involved at the two different scales (i.e., within and among species) because the mechanisms that lead to the fixation of specific alleles in species and their maintenance through time are certainly different from the selective forces acting within populations of a species. Indeed, we detected very little overlap between the sites identified among species in our study and the sites known to be involved at the intraspecific level [46]. Bridging the two evolutionary scales would require a much denser sampling within and across species, in order to track the fixation process of specific alleles and their divergence between closely related species.

Although we restricted our analysis to codons under positive selection so that the coevolutionary dynamics among genes in the melanocortin system can be more easily understood from the prism of adaptive and co-adaptive changes, it is difficult using our purely molecular approach to envision the rationale for the coevolutionary changes that the *MC1R* is inducing in other genes of the melanocortin system. Distinct hypotheses can explain why *MC1R* is inducing co-evolutionary changes in other genes of the melanocortin system although specific empirical studies will be needed to test how changes in the *MC1R* sequence influence other functional aspects of the melanocortin system. Regardless of the threshold used, we observed that *MC1R* induced coevolutionary changes mainly on the genes coding for the other melanocortin receptors (Table 1, Supplementary Materials Table S3). The fact that positive selection on the *MC1R* gene induced evolutionary responses on the other receptors supports the idea of modularity underlying complex adaptations based on multiple phenotypic aspects [47] Alternatively, it has been shown that the formation of MC1R-MC5R heterodimers is of functional relevance for the regulation of coloration in fish by altering MC1R sensitivity to its ligand, α-MSH [48] and similar heterodimers may exist between MC1R and, at least, MC3R [49]. The formation of such heterodimers may allow for coevolution among the melanocortin receptors, given that selection on the *MC1R* in relation, for instance, to adaptive coloration may require changes in the other receptors so that heterodimers can still be formed with the MC1R.

## 3. Material and Methods

### 3.1. Data Set

Our study comprises a total of 138 species representing the main vertebrate lineages (16 birds, 2 snakes, 7 lizards, 1 turtle, 1 monotreme, 3 marsupials, 81 placental mammals, 3 amphibians, 1 coelacanth, 20 teleost fish, 2 sharks, and 1 lamprey species: Supplementary Materials Figure S1). Complete coding sequences of the five melanocortin receptors (*MC1-5Rs*), the agonist ligand (*POMC*), antagonists (*ASIP* and *AGRP*), and convertases (*PCSK1* and *PCSK2*) were downloaded from Ensembl (http://www.ensembl.org) and NCBI (http://www.ncbi.nlm.nih.gov) databases on 02.04.2012 (see also Supplementary Materials Figure S1 and Table S2). The melanocortin receptors expanded early during vertebrate evolution, most likely as a consequence of two rounds of duplication, followed by a single gene duplication event that led to the MC5R [50]. The most ancient melanocortin receptor is suspected to belong to the sea and river lampreys (*MCa* and *MCb*, respectively) [50,51]. Some authors have proposed that *MCa* and *MCb* receptors represent two branches of the ancestral melanocortin receptors and that vertebrate *MC1R* and *MC2R* emerged from an ancestral MCa receptor, while *MCb* gave birth to the *MC3R* and a *MC4/5R* gene that later split into *MC4R* and *MC5R* [24,50]. Based on this interpretation, the lamprey’s *MCa* and *MCb* have been integrated as outgroups into the *MC1-2R* and *MC3-5R* data sets, respectively. Melanocortin receptors seem to have experienced duplication events followed by subfunctionalisation in certain fish species [50,52,53]. In those species with paralogous sequences, we excluded the sequence that was non-orthologous to the mammalian gene copies.

The *POMC* gene has been reported in invertebrates, suggesting that POMC-derived peptides probably appeared before the split between protostomes and deuterostomes [25,52,54,55]. Unfortunately, no sequences from invertebrates are available. Lampreys possess two copies of *POMC*, namely *POM* and *POC*, that we did not include into our data set because they likely experienced subfunctionalization [25]. We kept the paralog sequences of *Danio rerio*, *Gasterosteus aculeatu*, *Oreochromis niloticus* and *Oryzias latipes* species because they are fully functional copies of *POMC* that resulted from a latter duplication. Out of the 62 sequences available in Ensembl, 40 *POMC* sequences were finally kept for the analysis.

We translated the nucleotide sequence for the melanocortin receptors, *ASIP* and *AGRP* genes into amino acids before aligning them using the software Muscle [56]. Once aligned, we translated the sequences of amino acids back into nucleotide sequences for the analysis. The alignments of the coding sequences of the *POMC* and the convertases *PCSK1* and *PCSK2* were directly exported from Ensembl (ENSGT00390000016811, Euteleostomi.001). The alignments were filtered to exclude partial sequences and incomplete sequences. To perform the analyses of coevolution between pairs of genes, we concatenated the alignments of two genes from the same species (Supplementary Materials Table S2). Full sequences for all the 10 genes were available for only a few vertebrate species and, consequently, the number of genes varies from one species to another. *MC1R* is the most studied gene with a large number of sequences available across the vertebrates (Supplementary Materials Figure S1). However, the availability of *MC1R* sequences should not bias our results because the sampling level across vertebrates is consistent for each gene and all genes had sufficient numbers of sequences to have a correct power for the analyses performed [57]. For the coevolution analyses, we further reduced the concatenated alignments to include only the species that had both genes present in Ensembl (Supplementary Materials Table S2), which removed any effects of the larger sampling available for *MC1R*.

### 3.2. Phylogeny

We performed all analyses on the species tree of the organisms sampled (Supplementary Materials Figure S1). We constrained the topologies of the phylogenetic tree to follow the species tree from the Interactive Tree of Life (http://itol.embl.de/other_trees.shtml) and estimated, for each gene of the melanocortin system, the branch lengths of the species tree using the HKY85 + Gamma model as implemented in PhyML [58] from the alignments obtained from each gene. For our analyses, we used the topology of the species phylogenetic tree rather than the trees estimated from each gene. Our goal was to combine the estimation of selective pressure with the coevolution occurring between genes; thus, we needed to have a comparable topology for all our genes. Whenever possible (for the melanocortin receptors), we used the lamprey (*Lampetra fluviatilis*) as outgroup and a fish species when the sequence of a given gene was not available for the lamprey.

### 3.3. Estimation of Evolutionary Constraint and Selective Pressure

We measured the percentage of identity to evaluate the level of evolutionary conservatism of each gene of the melanocortin system. The nucleotide positions with a score higher than 95% were considered highly conserved positions. The reported percentage of identity for the alignment is the mean of the percentage of identity of all alignment positions. Although the species sampled are not identical between the studied genes, we took particular care to sample evenly the main vertebrate lineages so that the conservation scores can be compared between genes.

We used the MEME model [59] to classify each codon of the different melanocortin genes as either having evolved neutrally or under positive or purifying selection. The identification of selective pressures provided additional information that could not be measured by the percentage of identity. Purifying selection can be interpreted as an evolutionary force whose identification incorporates the phylogenetic relationship and the substitution process along the entire gene, whereas conservation is the description of a pattern observed in the alignment. We considered a maximum likelihood framework to fit a model allowing codons to be under selective pressure along branches of the phylogenetic tree. We compared this model based on the ten genes of the melanocortin system using likelihood ratio tests (LRT; *P*-value < 0.05) to a null model that did not allow the rate of non-synonymous substitutions to be larger than the rate of synonymous substitutions [59]. We controlled for multiple testing through false discovery rate, FDR, using the qvalue package in R [60]. We used the MEME model because we needed to determine the level of selection at codon sites assuming that episodic events of positive selection occurred throughout the evolution of vertebrates. We retrieved the LRT scores and their associated q-values for each codon position and determined whether they were under positive or purifying selection or whether they evolved neutrally. Mixed effects models of codon evolution [59] detected a statistically significant signal of selection or neutral evolution in approximately 59.5% of the codon sites (31.1% and 75.9% being the lowest and highest values, respectively; Figure 2b; Supplementary Materials Table S1). Within these unambiguous codon sites for each gene, we then calculated the proportion of codons that were under these different types of selective pressure for each gene.

We also counted the codons that were detected as evolving under positive selection with a probability higher than 95% and the proportion of branches of the vertebrate phylogeny where these codons were effectively inferred to be under positive selection. We used a Bayes factor greater than 1 to determine which branches should be counted in this procedure. Although a threshold of 1 for the Bayes factors might seem low, our goal here is not to identify the best model for each branch, but to compare the proportion of branches that show, for each gene, signs of positive selection. We did not consider the intensity of the selection (i.e., the exact level of the dN/dS ratio) in addition to the thresholds that are used to test whether the selection pressure is positive or purifying but counted only the codons that were significantly assigned to these types of selection. An accurate estimation of the parameter representing the dN/dS ratio can be difficult to obtain and it is better to assess this ratio using model comparisons (as describe above), which incorporated the uncertainty in the parameter value during the testing procedure.

### 3.4. Estimation of Coevolution

We used the maximum likelihood implementation of the model Coev [9] to estimate the nucleotide positions that were coevolving between pairs of genes. The analyses were based on the concatenated gene sequences and the species phylogenetic tree (Supplementary Materials Figure S1 and Table S2). We measured the score of coevolution for 219,556 pairs of positions for 45 combinations of inter-molecular alignments by comparing the fit of the Coev model against a null model of independent evolution. We characterized pairs of nucleotide positions as coevolving based on the difference in Akaike information criterion (ΔAIC) between the two models. However, assessing coevolution is difficult, and using the standard ΔAIC threshold for model selection can lead to spurious detection of coevolution [9,58]. We therefore used simulations to correct for bias in the evaluation of the ΔAIC distribution [9]. To confidently delimit the list of coevolving pairs for a given data set, we used the phylogenetic tree and branch lengths estimated from the concatenated alignments to simulate one alignment of 200 base pairs for each data set based on the independent JC69 model of substitution, as implemented in the package “evolver” from the PAML software [61]. For each pair of positions from the simulated alignments, we calculated the ΔAIC between the Coev model and the independent model that was used to simulate the data (i.e., JC69). We considered the 97.5th, 95th and 90th percentile values of these simulated ΔAIC distributions (i.e., one per pair of genes) as the thresholds to accept that two sites were coevolving. We used these three thresholds to assess the robustness of the coevolution measures and investigate the strength of the effect in our analyses. In total, we evaluated the score of coevolution of 40,000 simulated pairs of nucleotide positions for each inter-molecular analysis, which amounts to 1,800,000 pairs of positions analyzed.

### 3.5. Evolutionary Response in the Melanocortin System

We combined the predictions from the coevolution and selection analyses to estimate the extent to which positive selection exerted on a given site in a gene has resulted in coevolution of a site in a different gene. With this aim, we calculated for every gene the number of codons subject to positive selection that contained nucleotides inducing a coevolutionary response with another gene. For instance, among the 199 pairs of coevolving sites linking *MC2R* and *AGRP* (threshold 0.975, Supplementary Materials Table S4a), five were included in codons subject to positive selection in *AGRP* (Supplementary Materials Table S3a). Similarly, three nucleotides in *MC2R* induced an evolutionary response on *AGRP* and were contained in codons subject to positive selection (Supplementary Materials Table S3a). We used Principal Component Analysis (PCA) as implemented in the *prcomp* function in R [62] to summarize the evolutionary responses of the melanocortin genes.

## 4. Conclusions

Our study is a first step in the ultimate aim of considering all potential interactions between all genes within a genetic system. We show that the method developed by Dib et al. [8,9] allows to assess to what extent genes within functional modules such as the melanocortin system jointly evolve. Genes in the melanocortin system differ in their relative coevolutionary influence suggesting that, from this point of view, selection on a few aspects within the system can be of more importance than others for the evolution of the entire system. Future studies should consider potential co-evolutionary dynamics among the genes in the melanocortin system and the genes that also contribute to regulate the functions controlled by the melanocortin system. Despite the importance of the melanocortin system in regulating functions such as melanogenesis, energy homeostasis, or steroidogenesis, several other genes outside the melanocortin system can also influence these phenotypic aspects (e.g., several dozens of genes have been described to influence pigmentation in vertebrates [32]). The common participation of several genes in regulating a given function increases the likelihood for coevolution. Conducting these studies also offers the opportunity to test if co-evolution among genes that regulates the same function is, as intuition would suggest, stronger than among genes that have a common regulatory basis but code for different functions, such as those in the melanocortin system. In addition to these studies, replication in other known genetic networks or systems is required to assess the generality of our findings, particularly in systems with different evolutionary dynamics (for instance, with lower levels of sequence conservation). In contrast to other mechanisms such as regulation of gene expression, coevolution is not expected to be a major mechanism driving modular function [1]. However, we show that coevolution between genes in a genetic system can still be of certain importance. Future studies will be needed to assess whether coevolution among the genes within a system is higher than with genes belonging to a different one and try to disentangle the major factors triggering coevolution between genes. 

## Figures and Tables

**Table 1:** Coevolutionary response of the melanocortin system to selection on target genes. The table shows the number of nucleotides of a target gene that belong to a codon under positive selection (in rows) and induced a coevolutionary response in the other nine genes of the melanocortin system (in columns). For example in the Table 1, 10 nucleotides, which are part of a codon under positive selection in *MC1R*, induced a change in 10 sites in *MC5R* during vertebrate evolution. For each target gene, we give the number of pairs of sites that coevolved (note that a given site within a codon can be implicated in more than one pair of sites). We also provide the number of sites that belong to a codon under positive selection of a target gene that induced an evolutionary response in at least one of the other genes of the melanocortin system (this number can be smaller than the number of pairs because the same site can be involved in several pairs). Finally, we give the length in nucleotides of each human target gene and the percentage (in relation to this sequence length) of the number of different sites that belong to a codon under positive selection of a target gene and that induced an evolutionary response, which indicates the mean percentage of sites that coevolved with other genes of the melanocortin system. The sequences used in the coevolution and selection analyses where trimmed to remove conserved sites and regions of the alignment containing ambiguities. The length of the nucleotide sequence is therefore shorter than the total length given in Supplementary Materials Table S2. To evaluate the robustness of our method in assessing coevolution, we applied the ΔAIC threshold based on the 0.95 percentiles of the null distribution of ΔAIC obtained by simulations (the percentiles 0.975 and 0.90 are reported in Supplementary Materials Table S3). Whether the frequency of number of pairs or the frequency of number of sites per gene was significantly different than the frequency estimated for the rest of the genes in the melanocortin system was tested using Pearson’s χ^2^ tests corrected for multiple testing using the Benjamini-Hochberg approach. Genes with frequencies in the number of pairs or the number of sites significantly above the frequency for the rest of the genes are denoted with an ‘a’ superscript and those with frequencies below are denoted with a ‘b’ superscript. No superscript denotes non-significant differences.

		Coevolutionary Response to Positive Selection on Target Genes													
		*ASIP*	*AGRP*	*POMC*	*PCSK1*	*PCSK2*	*MC1R*	*MC2R*	*MC3R*	*MC4R*	*MC5R*	Nucleotide Sequence Length	Nb Pairs	Nb Different Sites	% Nb Different Sites
**Positive selection on target genes**	***ASIP***	-	4	1	6	6	3	14	9	2	3	396	176 ^a^	22 ^a^	5.6%
	***AGRP***	2	-	4	4	4	0	7	2	4	6	396	141 ^a^	9	2.3%
	***POMC***	0	2	-	2	0	0	3	0	0	1	801	33 ^b^	8	1.0%
	***PCSK1***	6	8	9	-	10	4	13	5	8	6	2256	322 ^b^	25 ^b^	1.1%
	***PCSK2***	3	1	0	10	-	3	6	2	4	3	1914	208 ^b^	13 ^b^	0.7%
	***MC1R***	5	3	1	1	3	-	11	8	7	13	951	311 ^a^	34 ^a^	3.6%
	***MC2R***	3	4	3	0	1	4	-	1	12	5	891	135	21	2.4%
	***MC3R***	0	3	0	0	3	8	12	-	5	3	969	209 ^a^	17	1.8%
	***MC4R***	0	2	0	0	2	5	3	1	-	0	996	85 ^b^	10	1.0%
	***MC5R***	6	8	2	4	3	15	3	3	2	-	972	192	30 ^a^	3.1%

**Table 2:** Number of coevolving nucleotide sites between pairs of genes of the melanocortin system using the ΔAIC thresholds based on the 0.95 percentile of the null distribution of ΔAIC obtained by simulation (see Methods). The percentiles 0.975 and 0.90 are given in Supplementary Materials Table S4.

	Coevolving Pairs										
	*ASIP*	*AGRP*	*POMC*	*PCSK1*	*PCSK2*	*MC1R*	*MC2R*	*MC3R*	*MC4R*	*MC5R*	Average
***ASIP***	-	181	101	519	170	643	913	631	105	323	398
***AGRP***	-	-	276	761	714	477	511	364	299	352	428
***POMC***	-	-	-	1348	-	606	682	104	340	192	405
***PCSK1***	-	-	-	-	4012	2672	3105	927	2704	1137	1909
***PCSK2***	-	-	-	-	-	2088	2207	1025	1410	1754	1487
***MC1R***	-	-	-	-	-	-	1253	1203	1636	1519	1344
***MC2R***	-	-	-	-	-	-	-	1336	1662	2011	1520
***MC3R***	-	-	-	-	-	-	-	-	1281	1454	925
***MC4R***	-	-	-	-	-	-	-	-	-	1250	1187
***MC5R***	-	-	-	-	-	-	-	-	-	-	1110

## Supplementary Materials

Supplementary materials can be found at www.mdpi.com/1422-0067/18/12/2618/s1.

## Availability of Data

The dataset supporting the conclusions of this article are available upon request.
