# Supplementary material for: Selection on the Major Color Gene Melanocortin-1-Receptor Shaped the Evolution of the Melanocortin System Genes
Source: Int J Mol Sci. 2017 Dec 5;18(12):2618. doi: 10.3390/ijms18122618 (PMC5751221; doi:10.3390/ijms18122618)
Supplement: Supplementary file 1 [file ijms-18-02618-s001.pdf]

Supplementary Information

Supplementary table 1. Evolutionary conservation and selection within ten genes of the melanocortin system in vertebrates. The conservation of the genes was measured as the percentage of identity and for completeness we report the number of sites with 95% conservation and proportion of conserved sites in the alignments. We used the MEME model to identify codons under neutral, positive, or purifying selection; these measures were obtained by considering only the codons that could be unambiguously assigned to one of these categories.

		Conservation			Selection				
Gene	♯ nucleotides in alignment	% of identity	Conserved nucleotide sites in alignment	% of conserved nucleotide sites in alignment	# codons analysed by MEME	# (%) assigned codons from MEME	% codons under positive selection	% of codons under purifying selection	% of codons that evolved neutrally
AGRP	585	74.0%	192	32.8%	195	88 (45.1%)	3.4%	88.6%	8.0%
ASIP	438	75.4%	114	26.0%	146	79 (54.1%)	6.3%	77.2%	16.5%
POMC	2'598	81.3%	115	4.4%	866	269 (31.1%)	3.0%	88.1%	8.9%
PCSK1	3'159	83.5%	838	26.5%	1'053	634 (60.2%)	1.6%	94.3%	4.1%
PCSK2	2'430	81.4%	795	32.7%	810	545 (67.3%)	2.2%	95.2%	2.6%
MC1R	1'083	76.7%	317	29.3%	361	206 (57.1%)	3.4%	89.8%	6.8%
MC2R	1'110	72.2%	323	29.1%	370	226 (61.1%)	2.2%	91.6%	6.2%
MC3R	993	80.0%	323	32.5%	331	247 (74.6%)	1.6%	92.7%	5.7%
MC4R	1'044	77.6%	436	41.8%	348	264 (75.9%)	0.4%	95.1%	4.6%
MC5R	1'005	76.3%	416	41.4%	335	230 (68.7%)	2.2%	93.9%	3.9%
**Mean**	**1'445**	**77.8%**	**386.9**	**29.7%**	**482**	**279**	**2.6%**	**90.7%**	**6.7%**

Supplementary Table 2. Characteristics of the intra-molecular alignments. For each gene pair, the number of sampled species, the size of the concatenated alignments, and the size of the concatenated alignment once conserved positions were filtered out (conservation score ≥ 0.9) are given.

Gene pair		Number of species	Intermolecular alignment size	Filtered intermolecular alignment size
AGRP	MC1R	17	529	431
AGRP	MC2R	34	644	597
AGRP	MC3R	25	471	363
AGRP	MC4R	33	527	451
AGRP	MC5R	31	593	543
ASIP	AGRP	30	317	283
ASIP	MC1R	26	546	428
ASIP	MC2R	28	661	555
ASIP	MC3R	24	488	395
ASIP	MC4R	31	544	403
ASIP	MC5R	25	610	519
PCSK1	AGRP	28	790	659
PCSK1	ASIP	22	807	541
PCSK1	MC1R	16	1019	883
PCSK1	MC2R	27	1134	1080
PCSK1	MC3R	19	961	667
PCSK1	MC4R	26	1017	957
PCSK1	MC5R	23	1083	984
PCSK2	AGRP	28	672	632
PCSK2	ASIP	22	689	561
PCSK2	PCSK1	27	1162	1136
PCSK2	MC1R	17	901	785
PCSK2	MC2R	29	1016	995
PCSK2	MC3R	21	843	614
PCSK2	MC4R	30	899	885
PCSK2	MC5R	27	965	934
MC2R	MC1R	22	873	805
MC3R	MC1R	19	700	580
MC3R	MC2R	25	815	634
MC4R	MC1R	29	756	695

MC4R	MC2R	41	871	850
MC4R	MC3R	29	698	570
MC5R	MC1R	17	822	756
MC5R	MC2R	32	927	908
MC5R	MC3R	24	754	676
MC5R	MC4R	36	810	808
POMC	AGRP	30	281	281
POMC	ASIP	21	298	206
POMC	PCSK1	27	771	771
POMC	PCSK2	30	653	653
POMC	MC1R	17	510	487
POMC	MC2R	28	625	623
POMC	MC3R	17	452	326
POMC	MC4R	30	508	508
POMC	MC5R	27	574	571

Supplementary table 3. Coevolutionary response of the melanocortin system to selection on target genes. The table shows the number of nucleotides of a target gene that belong to a codon under positive selection (in rows) and induced a coevolutionary response in the other nine genes of the melanocortin system (in columns). For example in the table A, 10 nucleotides, which are part of a codon under positive selection in *MC1R*, induced a change in 10 sites in *MC5R* during vertebrate evolution. For each target gene, we give the number of pairs of sites that coevolved (note that a given site within a codon can be implicated in more than one pair of sites). We also provide the number of sites that belong to a codon under positive selection of a target gene that induced an evolutionary response in at least one of the other genes of the melanocortin system (this number can be smaller than the number of pairs because the same site can be involved in several pairs). We finally give the length in nucleotides of each human target gene and the percentage (in relation to this sequence length) of the number of different sites that belong to a codon under positive selection of a target gene and that induced an evolutionary response, which indicates the mean percentage of sites that coevolved with other genes of the melanocortin system. The sequences used in the coevolution and selection analyses where trimmed to remove conserved sites and regions of the alignment containing ambiguities. The length of the nucleotide sequence is therefore shorter than the total length given in Supplementary table 1. To evaluate the robustness of our method in assessing coevolution, we applied three ΔAIC thresholds based on the 0.975 (A), 0.95 (table 1) and 0.90 (B) percentiles of the null distribution of ΔAIC obtained by simulations. Whether the frequency of number of pairs or the frequency of number of sites per gene was significantly different than the frequency estimated for the rest of the genes in the melanocortin system was tested using Pearson's χ^2 tests corrected for multiple testing using the Benjamini-Hochberg approach. Genes with frequencies in the number of pairs or the number of sites significantly above the frequency for the rest of the genes are denoted with an 'a' superscript and those with frequencies below are denoted with a 'b' superscript. No superscript denotes non-significant differences.

Supplementary table 3A) Threshold 0.975

		Coevolutionary response to positive selection on target genes													
		ASIP	***AGRP***	***POMC***	***PCSK1***	***PCSK2***	***MC1R***	***MC2R***	***MC3R***	***MC4R***	***MC5R***	**Nucleotide sequence length**	**Nb pairs**	**Nb different sites**	**% Nb different sites**
**Positive selection on target genes**	***ASIP***	-	3	0	0	5	1	8	5	0	0	396	39[a]	15[a]	3.8%
	AGRP	1	-	3	3	2	0	5	2	0	0	396	25[a]	8	2.0%
	POMC	0	0	-	0	0	0	3	0	0	0	801	6[b]	3[b]	0.4%
	PCSK1	0	4	4	-	4	3	11	3	0	0	2'256	67[b]	19	0.8%
	PCSK2	3	0	0	4	-	3	5	2	0	0	1'914	32[b]	9[b]	0.5%
	MC1R	2	1	0	1	2	-	5	5	4	10	951	74[a]	24[a]	2.5%
	MC2R	2	3	2	0	0	8	-	1	11	4	891	67[a]	21[a]	2.4%
	MC3R	0	0	0	0	3	8	11	-	2	1	969	70[a]	15	1.5%
	MC4R	0	0	0	0	0	3	3	1	-	0	996	21[b]	5	0.5%
	MC5R	0	0	0	0	0	8	1	1	0	-	972	27	10	1.0%

Supplementary table 3B) Threshold 0.90

		Coevolutionary response to positive selection on target genes													
		ASIP	*AGRP*	*POMC*	*PCSK1*	*PCSK2*	*MC1R*	*MC2R*	*MC3R*	*MC4R*	*MC5R*	**Nucleotide sequence length**	**Nb pairs**	**Nb different sites**	**% Nb different sites**
**Positive selection on target genes**	***ASIP***	-	6	3	6	8	3	14	9	11	4	396	452[a]	22[a]	5.6%
	AGRP	3	-	5	4	4	0	7	2	4	6	396	232[a]	9	2.3%
	POMC	0	2	-	2	0	0	3	1	0	3	801	53[b]	11	1.4%
	PCSK1	7	8	11	-	10	5	14	6	8	9	2'256	649[b]	25[b]	1.1%
	PCSK2	4	1	0	10	-	3	6	2	5	3	1'914	402[b]	14[b]	0.7%
	MC1R	5	3	1	1	4	-	14	8	7	15	951	664[a]	37[a]	3.9%
	MC2R	4	4	3	0	1	15	-	1	13	6	891	283	28[a]	3.1%
	MC3R	0	3	0	0	3	8	13	-	6	3	969	399[a]	17	1.8%
	MC4R	0	2	0	0	2	5	3	1	-	0	996	115[b]	10[b]	1.0%
	MC5R	7	9	3	5	4	22	3	3	2	-	972	431[a]	38[a]	3.9%

Supplementary table 4. Number of coevolving nucleotide sites between pairs of genes of the melanocortin system using the ΔAIC thresholds based on the 0.975 (A) and 0.90 (B) percentile of the null distribution of ΔAIC obtained by simulation (see Methods). The percentile 0.95 is presented in table 2 in the main text.

A) Threshold 0.975

	Coevolving pairs										
	ASIP	***AGRP***	***POMC***	***PCSK1***	***PCSK2***	***MC1R***	***MC2R***	***MC3R***	***MC4R***	***MC5R***	**Average**
ASIP		48	44	3	120	124	294	147	0	1	87
AGRP			88	221	348	139	199	62	1	0	122
POMC				294	0	156	273	31	0	31	102
PCSK1					579	541	1045	129	14	21	316
PCSK2						349	858	191	0	38	276
MC1R							238	200	209	442	266
MC2R								292	805	1074	564
MC3R									3	453	168
MC4R										1	115
MC5R											229

B) Threshold 0.90

	Coevolving pairs										
	ASIP	***AGRP***	***POMC***	***PCSK1***	***PCSK2***	***MC1R***	***MC2R***	***MC3R***	***MC4R***	***MC5R***	**Average**
ASIP	-	434	152	1316	1356	1900	1487	1016	614	1711	1110
AGRP	-	-	473	1227	1057	1458	851	709	556	827	812
POMC	-	-	-	2715	-	1700	1087	553	693	237	846
PCSK1	-	-	-	-	6239	6578	5357	3033	4751	3164	3820
PCSK2	-	-	-	-	-	4708	3191	1950	2669	3739	2768
MC1R	-	-	-	-	-	-	3284	3642	2550	3474	3255
MC2R	-	-	-	-	-	-	-	2365	2146	2753	2502
MC3R	-	-	-	-	-	-	-	-	1871	2318	1940
MC4R	-	-	-	-	-	-	-	-	-	1880	1970
MC5R	-	-	-	-	-	-	-	-	-	-	2234

Supplementary table 5. Percentage of branches of the vertebrate phylogenetic tree where genes of the melanocortin system coevolved as a result of positive selection. Rows represent the proportion of branches where positive selection exerted on a target gene induced an evolutionary response on genes written in columns.

		Co-evolutionary response to positive selection on target genes										
		ASIP	***AGRP***	***POMC***	***PCSK1***	***PCSK2***	***MC1R***	***MC2R***	***MC3R***	***MC4R***	***MC5R***	**Mean**
**Proportion of branches under positive selection**	***ASIP***	-	5.3%	0	0	12.7%	20.4%	14.2%	16.1%	0	0	7.6%
	AGRP	5.3%	-	9.4%	15.7%	11.8%	0	13.8%	4.3%	0	0	6.1%
	POMC	0	0	-	0	0	0	13.3%	0	0	0	1.5%
	PCSK1	0	7.9%	10.0%	-	3.4%	11.0%	9.7%	16.2%	0	0	6.5%
	PCSK2	8.1%	0	0	25.7%	-	19.4%	25.7%	17.9%	0	0	9.9%
	MC1R	6.1%	12.9%	0	10.3%	12.9%	-	11.3%	17.5%	12.3%	14.4%	10.2%
	MC2R	8.3%	11.5%	22.4%	0	0	14.4%	-	4.3%	11.4%	13.1%	8.6%
	MC3R	0	0	0	0	5.8%	10.1%	12.2%	-	3.6%	8.9%	4.5%
	MC4R	0	0	0	0	0	17.5%	18.4%	18.2%	-	0	6.0%
	MC5R	0	0	0	0	0	14.9%	4.9%	2.2%	0	-	2.4%

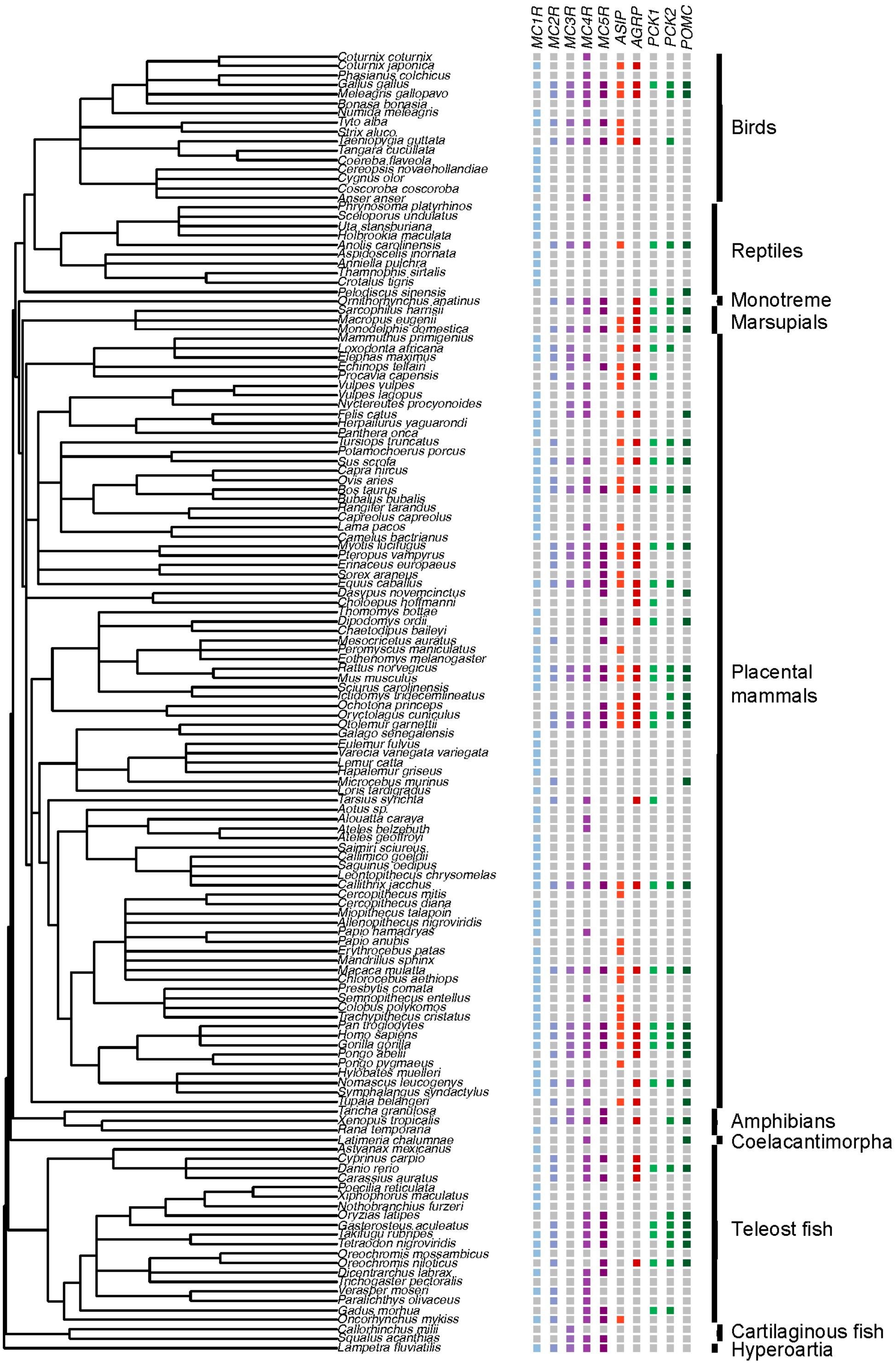

Supplementary Figure 1. Sequences sampled across the vertebrate phylogenetic tree. On the left are the species used and the phylogenetic relationships among them used in this study. The columns on the right represent sequence availability for each species and gene of the melanocortin system (grey: unavailable, colored: available).

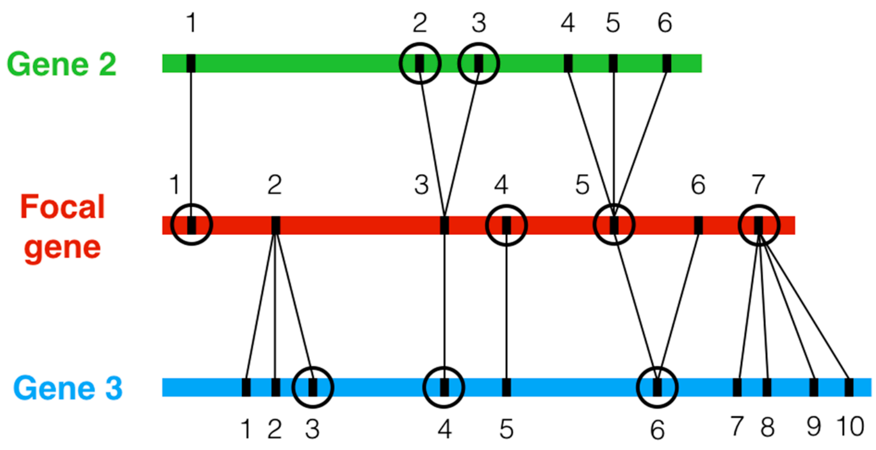

Supplementary figure 2. Schematic presentation of indices of coevolutionary processes. i) For each focal gene, we counted the total number of pairs identified as coevolving with the other genes of the melanocortin system (Nb coevolving pairs in figure 3; note that these sites did not necessarily evolve under positive selection); here, the focal gene has six pairs of coevolving sites with Gene 2 and 11 pairs with Gene 3 and for the analysis we calculated the grand total (6 + 11). ii) Because a given site can be involved in multiple pairs, for each gene we counted the number of sites coevolving with the other genes (Nb sites in figure 3); here, the focal gene has 7 sites. iii) We calculated the percentage of sites (% coevolving sites in figure 2) that coevolved with the other genes; here 7 sites divided by the total number of nucleotide sites of the focal gene. iv) For each focal gene, we determined how many sites in a codon under positive selection coevolved with other sites of the other genes (Nb pairs under selection in figure 3); the focal gene has two sites in codons under positive selection (as indicated by the circles) that coevolved with at least one site of Gene 2 and three sites in codons under positive selection that coevolved with at least one site of Gene 3. The total number of sites in codons under positive selection on the focal gene that induces a coevolutionary response in the other genes is four. v) To measure whether the effect of positive selection exerted on a focal gene was homogeneously distributed on the other genes, we calculated the coefficient of variation (CV in figure 3) of the number of nucleotide sites in

codons under positive selection in the focal gene and that induced a change in at least one nucleotide site of the other genes; 2 and 3 sites in codons under positive selection of the focal gene induced changes in the nucleotide sequences of Gene 2 and Gene 3, respectively. vi). Each gene had a different number of nucleotide sites (sequence length in figure 3). vii) For each gene, we calculated the percentage of branches along the phylogenetic tree that contained sites in codons under positive selection that induced coevolution in sites of other genes of the melanocortin system.
